# Fulminant myocarditis during postoperative adjuvant chemotherapy for lung cancer with atezolizumab: a case report

**DOI:** 10.1186/s13256-024-04447-w

**Published:** 2024-03-16

**Authors:** Takuya Tokunaga, Masaya Aoki, Koki Maruyama, Yuto Nonaka, Kota Kariatsumari, Koichi Sakasegawa, Kazuhiro Ueda

**Affiliations:** 1 Department of General Thoracic Surgery, Kagoshima City Hospital, 37-1, Uearatacho, Kagoshima, 890-8760 Japan; 2 Department of General Thoracic Surgery, Kagoshima University Graduate School of Dental and Medical Science, 8-35-1 Sakuragaoka, Kagoshima, 890-8520 Japan

**Keywords:** Fulminant myocarditis, Lung cancer, Adjuvant chemotherapy, Atezolizumab

## Abstract

**Background:**

Postoperative adjuvant systemic therapy with atezolizumab for lung cancer has been reported to be effective. Although myocarditis is a rare immune adverse event associated with atezolizumab, it can have a serious course and should be treated with caution. We herein report a case of fulminant myocarditis during adjuvant systemic therapy with atezolizumab.

**Case presentation:**

The patient was a 49-year-old Asian woman. She was diagnosed with pT2aN1M0 stage IIB (Programmed Death Ligand 1(PD-L1), 50%) after surgery for right upper lobe lung adenocarcinoma. Atezolizumab was administered following platinum-based adjuvant chemotherapy. On day 14, the patient was hospitalized because of deterioration in her general condition caused by fever. On day 16, she developed dyspnea, which worsened, and on day 17, she experienced shock. Blood tests, echocardiography, and cardiac catheterization were performed, and the patient was diagnosed with cardiogenic shock due to myocarditis. Initial measures did not improve the patient’s shock state. The patient was transferred to hospital for the use of an assistive circulatory system. Pulse steroid therapy was administered, and myocarditis showed a tendency toward improvement. A retrospective review of the patient’s history revealed a decreased lymphocyte count and an increase in the neutrophil/lymphocyte ratio, which may be useful for detecting severe immune-related adverse events. The troponin levels were elevated, but creatine phosphokinase level remained within the normal range.

**Conclusion:**

Myocarditis can be fatal due to the rapid progression of symptoms. Close follow-up, a prompt diagnosis, and therapeutic intervention are important. Decreased lymphocyte counts, increased neutrophil/lymphocyte ratios, and the measurement of multiple myocardial biomarkers are considered useful for the early diagnosis of myocarditis.

## Background

Immune-related adverse events (irAEs) associated with immune checkpoint inhibitors (ICIs) include myocarditis, which is rare but can have a rapid course. Although myocarditis has been reported with atezolizumab [1], there are no reports of myocarditis associated with postoperative adjuvant systemic therapy for lung cancer. We herein describe a case of fulminant myocarditis that developed during adjuvant systemic therapy with atezolizumab, and report on the associated markers.

## Case presentation

A 49-year-old Asian woman underwent video-assisted thoracic surgery for right upper lobe lung adenocarcinoma and was diagnosed with pT2aN1M0 stage IIB (tumor size 16 mm, pleural invasion+, PD-L1 Tumor Proportion Score (TPS), 50%). There was no previous or family history of cardiac disease, and no abnormal findings in the cardiac function were detected before surgery. The patient had a history of hypertension, fatty liver disease, and chronic thyroiditis. The patient received a total of three courses of adjuvant chemotherapy. Initially, treatment with cisplatin and vinorelbine was started. However, it was discontinued after one course due to Common Terminology Criteria for Adverse Events (CTCAE) (v5.0) grade 4 neutropenia, and 6 weeks after the initiation of adjuvant chemotherapy, the drugs were changed to carboplatin and paclitaxel, which were administered for two courses, but were discontinued due to CTCAE (v5.0) grade 2 dysesthesia. Then, 10 weeks after the completion of the platinum doublet, administration of atezolizumab was started as postoperative adjuvant systemic therapy. There was no history of autoimmune disease, active hepatitis B, hepatitis C, active tuberculosis, idiopathic pulmonary fibrosis, organizing pneumonia, drug-induced pneumonia, or idiopathic pneumonia. Additionally, no abnormal findings were observed in general blood biochemical tests. Therefore, we determined that it was reasonable to administer atezolizumab to this patient. The patient was hospitalized and followed up from day 1, and was discharged from the hospital on day 8 without any major problems.

**Fig. 1:**
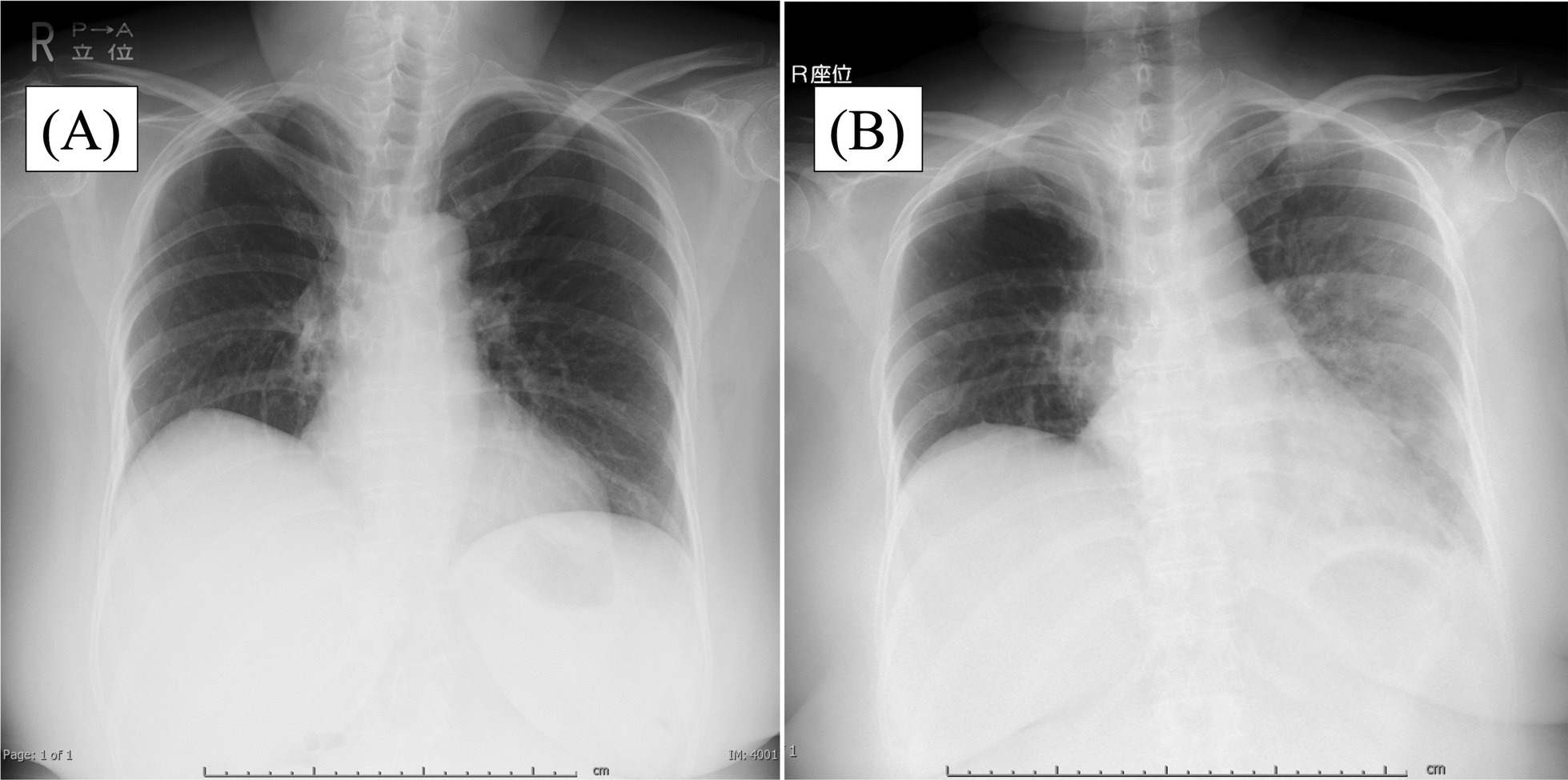
Chest radiograph at admission revealing no decreased permeability of the lung fields (**a** day 13). Chest radiograph at the onset of myocarditis revealed decreased permeability of the lung fields (**b** day 17)

On day 9, fever (38 °C) appeared and persisted, and the patient visited our department on day 13. She was admitted to our department for symptomatic treatment of persistent fever and appetite loss. Upon arrival at the hospital, the patient was conscious, her body temperature was 40.3 °C, her blood pressure was 134/80 mmHg, and her pulse was 116 beats per minute. Blood tests revealed elevated hepatic enzyme levels. Table 1A presents the blood test findings at our department’s visit. Chest radiography did not demonstrate any obvious findings (Fig. 1A). Computed tomography (CT) revealed an enlarged and painful right cervical lymph node. Blood tests showed elevated C-reactive protein (CRP) levels, and subacute necrotizing lymphadenitis was diagnosed. On day 16, dyspnea on exertion worsened, and dyspnea at rest appeared on day 17. Chest radiography performed on the same day showed pulmonary congestion (Fig. 1B). Her systolic blood pressure was less than 90 mmHg, and she was in a state of shock. She was admitted to the intensive care unit (ICU). A blood test showed aspartate aminotransferase (AST) 86U/L, alanine aminotransferase (ALT) 53U/L, lactate dehydrogenase (LDH) 566U/L, and troponin I 538.8 pg/mL, with a significant increase in the myocardial enzyme level (Table 1B). After admission to the ICU, the patient was suspected to have cardiac disease because of elevated troponin I levels on blood tests. Electrocardiography, echocardiography, cardiac catheterization, and myocardial biopsy were also performed. Electrocardiography revealed tachycardia (125 beats per minute). Echocardiography revealed circumferential wall hypokinesia and pericardial effusion. Cardiac catheterization revealed cardiac index (CI) 2.05 L/m^2^ and right ventricular pressure (RVP) 35 mmHg (Forrester type 4). Coronary angiography was negative for myocardial infarction. On the basis of these findings, cardiogenic shock due to myocarditis was diagnosed. The patient needed to be intubated and supported via mechanical ventilation and treated with a combination of cardioactive drugs (dobutamine 3 μg/kg/minute, noradrenaline 0.2 μg/kg/minute, and adrenaline 0.15 μg/kg/minute). The patient also had an intra-aortic balloon pump placed via the left femoral artery to assist in circulatory support, however, she remained hypotensive. The patient was transferred to the cardiology department of another hospital on the same day for the use of an assistive circulatory device.

**Table 1:** Blood test findings

(A)	Normal range	(B)	Normal range
White blood cell (/μl)	4300 (3300–8600)	White blood cell (/μl)	6900 (3300–8600)
Lymph (%)	18.5 (25.0–45.0)	Lymph (%)	23 (25.0–45.0)
Hemoglobin (g/dl)	12.0 (11.6–14.8)	CK (U/L)	130 (41–153)
ALT (U/L)	27 (7–23)	CK-MB (ng/ml)	3 (0–10)
AST (U/L)	23 (13–30)	Troponin I (pg/ml)	538.8 (0.0–26.2)
LDH (U/L)	285 (124–222)	BNP (pg/ml)	503.0 (0.0–18.4)
CRP (mg/dl)	4.76 (0.00–0.14)	Myoglobin (ng/ml)	200 (0–116)
BUN (mg/dl)	0.89 (8.0–22.0)		
Cre (mg/dl)	0.83 (0.46–0.79)		
Na (mmol/dl)	136 (138–145)		
K (mmol/dl)	4.5 (3.6–4.8)		
Cl (mmol/dl)	99 (101–108)		
Ca (mg/dl)	9.0 (8.8–10.1)		
FT4 (ng/dl)	0.89 (0.70–1.48)		
FT3 (pg/ml)	2.16 (1.68–3.67)		
TSH (μIU/mL)	2.05 (0.61–4.23)		

*ALT* alanine aminotransferase, *AST* aspartate aminotransferase, *LDH* lactate dehydrogenase, *CRP* Creactive protein, *BUN* blood urea nitrogen, *Cre* creatinine, *FT4* free thyroxine, *FT3* free triiodothyronine, *TSH* thyroid stimulating hormone, *CK* creatine phosphokinase, *CK-MB* creatine kinase MB, *BNP* brain natriuretic peptide

**Fig. 2:**
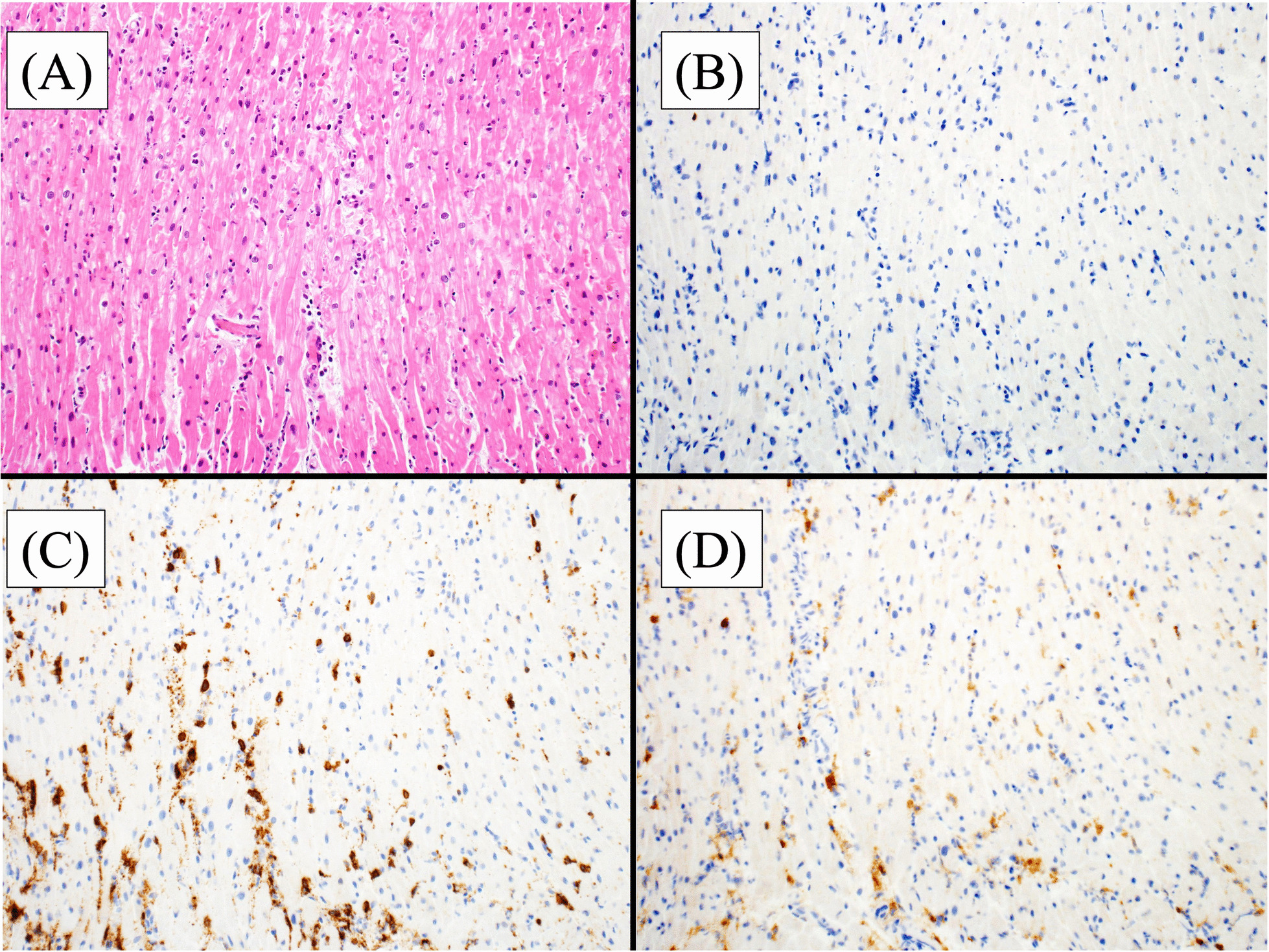
Myocardial biopsy specimen showing the dense infiltration of neutrophils and lymphocytes, mainly in the interstitium (**a** hematoxylin and eosin, x50). Immunohistochemically, there are few CD79a-positive B cells in the tissue (**b** CD79a x50). In infiltrating T cells, CD8-positive cells are abundant (**c** CD8 x50) and CD4-positive cells are minimal (**d** CD4 x50)

**Fig. 3:**
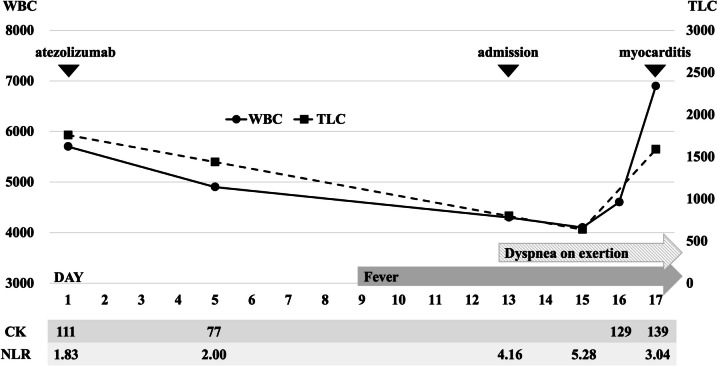
From the date of admission to the date of onset of myocarditis. The patient developed myocarditis during hospitalization and was admitted to the ICU on day 17 of hospitalization. CK levels remained within the normal range from the start of treatment until the onset of myocarditis, TLC showed the greatest decrease before the onset of myocarditis, and NLR showed the greatest increase before the onset of myocarditis. *WBC* white blood cell, *TLC* total lymphocyte count, *CK* creatine kinase, *NLR* neutrophil/lymphocyte ratio

The patient underwent placement of an IMPELLA, which is a percutaneous cardiopulmonary support device. The patient was treated with pulse steroid therapy (methylprednisolone 1000 mg, days 17–19). The patient was weaned from the ventilator on day 24 and was discharged home on day 71. All tested viral antibodies were negative. A myocardial biopsy showed myocardial infiltration of CD8-positive cells (Fig. 2). These results were consistent with myocarditis caused by irAEs. The course from the introduction of atezolizumab to the onset of myocarditis is shown in Fig. 3. Retrospectively, total lymphocyte count (TLC) decreased and the neutrophil/lymphocyte ratio (NLR) increased before the onset of myocarditis, and creatine phosphokinase (CK) remained in the normal range.

More than 1 year has passed since atezolizumab was discontinued, and no increase in cardiac troponin or recurrence of lung cancer has been observed.

## Discussion

The Impower010 study reported the efficacy of atezolizumab as postoperative adjuvant systemic therapy [2]. Since general thoracic surgeons are increasingly using ICIs as adjuvant systemic therapy, it is important to detect serious side effects at an early stage.

Myocarditis as an irAE with ICIs was reported to be 0.09–1.14% [3, 4]. When used as adjuvant systemic therapy with atezolizumab, the frequency was reported to be 0.2% [5]. According to Mahmood [3], the median onset of myocarditis is 34 days after the administration of ICI therapy, and approximately 81% of patients develop the disease within 3 months of starting treatment. The onset of myocarditis in this patient also occurred within 3 months after the start of treatment. Myocarditis tended to appear relatively early after the initiation of treatment. The mechanism underlying the development of myocarditis is thought to involve the destruction of peripheral immune tolerance mechanisms by PD-L1 expressed in cardiomyocytes and vascular endothelial cells. These peripheral immune tolerance mechanisms are disrupted by ICI administration, leading to lymphocyte attack on the myocardium [6]. The fatality rate of myocarditis is said to be 25–50% [7]. Myocarditis presents with a wide range of clinical manifestations, ranging from asymptomatic to cardiogenic shock and death, and requires careful monitoring.

Myocardial troponin levels are reported to be abnormal in approximately 94% of patients with ICI-associated myocarditis [4], and were also elevated in this case. The Atezolizumab Appropriate Use Guide [1] recommends the observation of chest pain, elevated CK, and abnormal electrocardiograms in patients with myocarditis. In addition, when myocarditis is suspected, multiple myocardial biomarker measurements should be considered, including the measurement of myocardial troponin levels, even when CK is within the normal range. Viral myocarditis should be considered as a differential disease. In this case, the tests for the viruses submitted were negative, including influenza virus, severe acute respiratory syndrome coronavirus 2 (SARS-CoV-2), cytomegalovirus, coxsackie virus, adenovirus, Herpes simplex virus, and Epstein–Barr virus. In this context, myocarditis was considered an adverse effect of ICI.

The association of TLC and NLR with the prognosis has long been observed, and Sacdalan [8] reported that a higher NLR contributes to a lower response rate to ICI treatment for lung cancer. A higher TLC at initiation [9] and a lower NLR at initiation [10] are reported to be associated with an increased incidence of irAEs. In addition to the starting values, Fujisawa [11] and Drobni [12] reported that a decrease in the TLC and an increase in the NLR were associated with the development of irAEs. Fujisawa [11] reported that a ≥ 32% decrease in the TLC was associated with a fivefold increase in the incidence of irAEs. A possible mechanism by which decreased lymphocyte counts are associated with the development of irAEs is a decrease in peripheral blood lymphocyte counts due to the mobilization of lymphocytes into tissues. In viral infections, lymphocyte counts in the peripheral blood decrease due to the temporary mobilization of lymphocytes to lymphoid tissues and inflamed organs. It is not surprising that a similar condition may occur with irAEs. In this case, TLC decreased, and NLR increased from the start of ICI administration to the onset of disease, while TLC decreased by approximately 64%. From a retrospective viewpoint, this patient should be followed up carefully due to the possible development of severe irAEs, including myocarditis. If the onset of fulminant myocarditis is detected during outpatient follow-up, it may be too late to save the patient’s life. By careful observation of changes in TLC and NLR during routine clinical practice, we may be able to predict the onset of fulminant myocarditis at an earlier stage.

In cases of myocarditis with CTACE grade ≥ 3, pulse steroid therapy (methylprednisolone 1 g per day for 3 days) is an option; however, its usefulness has not been established beyond myocarditis caused by ICI therapy, so each case must be treated on a case-by-case basis. Because of the rapid progression of the condition, there is a high possibility that an assistive circulatory system will be required, and collaboration between the cardiology department and intensive care unit is essential.

## Conclusion

We reported a case of fulminant myocarditis caused by atezolizumab. A decrease in TLC and an increase in NLR are considered useful for detecting severe irAEs. When the onset of irAEs is suspected, it is important to perform multiple marker measurements, electrocardiography, and echocardiography, as well as to collaborate with other departments for prompt diagnosis and treatment.

## Data Availability

The datasets supporting the conclusions of this article are included within the article.

## Abbreviations

irAEs: Immune-related adverse events
ICIs: Immune checkpoint inhibitors
TLC: Total lymphocyte count
NLR: Neutrophil/lymphocyte ratio
CT: Computed tomography
CRP: C-reactive protein
